# Lipoprotein(a) as a Causal Risk Factor for Cardiovascular Disease

**DOI:** 10.1007/s12170-025-00760-1

**Published:** 2025-02-18

**Authors:** Sean Doherty, Sebastian Hernandez, Rishi Rikhi, Saeid Mirzai, Chris De Los Reyes, Scott McIntosh, Robert C. Block, Michael D. Shapiro

**Affiliations:** 1 Department of Internal Medicine, Wake Forest University School of Medicine, Medical Center Boulevard, Winston-Salem, NC 27157 USA; 2 Center for Prevention of Cardiovascular Disease, Section on Cardiovascular Medicine, Department of Internal Medicine, Medical Center Boulevard, Wake Forest University School of Medicine, Medical Center Boulevard, Winston-Salem, NC 27157 USA; 3 Department of Public Health Sciences, University of Rochester School of Medicine and Dentistry, 14642 Rochester, NY USA; 4 Department of Public Health Sciences, Division of Public Health Sciences, University of Rochester Medical Center, Rochester, NY 14642 USA; 5 Department of Public Health Sciences, Division of Epidemiology, Department of Medicine’s Cardiology Division, University of Rochester School of Medicine and Dentistry, Rochester, NY 14642 USA

**Keywords:** Lipoprotein(a), Low-density lipoprotein cholesterol, Atherosclerotic cardiovascular disease, Calcific aortic valve stenosis

## Abstract

**Purpose of Review:**

Lipoprotein(a) [Lp(a)], an atherogenic low-density lipoprotein cholesterol (LDL-C)-like molecule, has emerged as an important risk factor for the development of atherosclerotic cardiovascular disease (ASCVD). This review summarizes the evidence supporting Lp(a) as a causal risk factor for ASCVD and calcific aortic valve stenosis (CAVS).

**Recent Findings:**

Lp(a) is largely (~ 90%) genetically determined and approximately 20% of the global population has elevated Lp(a). The unique structure of Lp(a) leads to proatherogenic, proinflammatory, and antifibrinolytic properties. Data from epidemiological, genome-wide association, Mendelian randomization, and meta-analyses have shown a clear association between Lp(a) and ASCVD, as well as CAVS. There are emerging data on the association between Lp(a) and ischemic stroke, peripheral arterial disease, and heart failure; however, the associations are not as strong.

**Summary:**

Several lines of evidence support Lp(a) as a causal risk factor for ASCVD and CAVS. The 2024 National Lipid Association guidelines, 2022 European Atherosclerosis Society, and 2021 Canadian Cardiology Society guidelines recommend testing Lp(a) once in all adults to guide primary prevention efforts. Further studies on cardiovascular outcomes with Lp(a) targeted therapies will provide more insight on causal relationship between Lp(a) and cardiovascular disease.

## Introduction

Lipoprotein(a) [Lp(a)], first described in 1963 by Norwegian physician Kåre Berg, is a low-density lipoprotein cholesterol (LDL-C)-like particle whose apolipoprotein B100 (apoB) moiety is covalently linked to a polymorphic glycoprotein known as apolipoprotein(a) [apo(a)] [1]. The apo(a) portion of Lp(a) has structural similarities to plasminogen, though it is truncated, containing only kringles IV and V domains and an inactive protease domain [2, 3]. Moreover, the kringle IV domain of apo(a) has 10 subtypes, with copy number variation of kringle IV subtype 2 (KIV_2_) [4]. Additionally, Lp(a) is the primary carrier of oxidized phospholipids, promoting vascular inflammation [5, 6]. Finally, since apo(a) shares homology with plasminogen, it is thought to contribute to the antifibrinolytic potential of Lp(a) by competitively blocking the conversion of plasminogen to plasmin [2, 7, 8]. Thus, Lp(a) has antifibrinolytic and proinflammatory properties, with data suggesting Lp(a) to be six times more atherogenic than LDL-C [9].

Elevated Lp(a) is defined by the 80th population percentile, roughly ≥ 50mg/dL or ≥ 125nmol/L. Unlike LDL-C which has a roughly normal distribution in the population, Lp(a) is highly skewed. Moreover, ~ 70–90% of plasma levels are genetically determined [10–13]. Higher Lp(a) levels have been observed among Black populations, with one study finding median levels of 75 nmol/L compared to 31, 19, and 16 nmol/L for South Asian, White and East Asian populations, respectively [14]. Post-menopausal women also tend to have higher Lp(a) concentrations with median levels of 22 nmol/L compared to 17 nmol/L in men [14]. While Lp(a) concentrations vary across ethnic groups worldwide, the associated atherosclerotic cardiovascular disease (ASCVD) risk remains similar across populations [14–23].

Advances in measurement techniques have clarified the relationship between Lp(a) and cardiovascular disease (CVD). Early studies from the 1990s using isoform-sensitive assays, which overestimated Lp(a) concentration in individuals with large KIV_2_ domains [24], failed to establish significant associations between Lp(a) and the risk of ASCVD or myocardial infarction (MI) [1, 24]. However, more recent research has consistently shown that elevated Lp(a) levels are associated with increased risk of ASCVD and calcific aortic valve stenosis (CAVS) [25, 26]; these studies have relied on the newer ELISA assays that measure Lp(a) concentrations independent of isoform size [24].

This review synthesizes the growing body of epidemiological, genetic, and mechanistic evidence that establishes Lp(a) as a causal risk factor for CVD, with particular emphasis on its role in ASCVD and CAVS.

## Lipoprotein(a) as a Risk Factor for ASCVD

### In-Vitro and Animal Studies

**Fig. 1:**
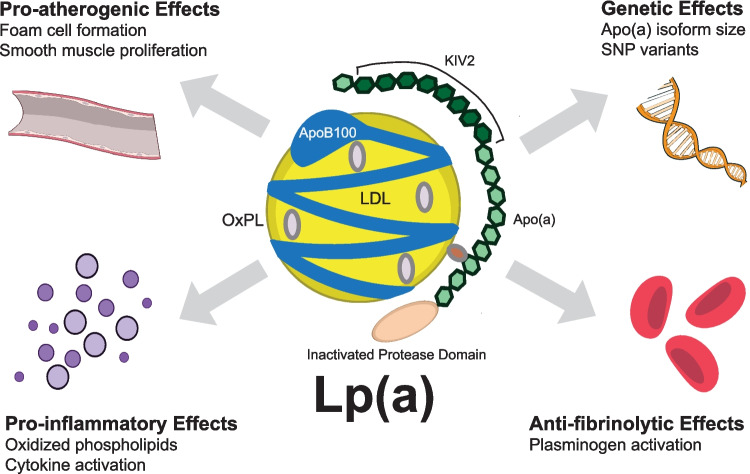
Mechanisms Linking Elevated Lp(a) with ASCVD. Abbreviations: Apo(a), apolipoprotein(a); ApoB100, apolipoprotein B-100; ASCVD, atherosclerotic cardiovascular disease; KIV2, kringle IV subtype 2; LDL, low-density lipoprotein; Lp(a), lipoprotein(a); OxPL, oxidized phospholipids; SNP, single-nucleotide polymorphism

In vitro and animal studies have shown that elevated Lp(a) levels contribute to CVD risk through mechanisms such as foam cell formation, smooth muscle cell proliferation, plaque inflammation, and plaque instability, all of which drive atherosclerotic progression and plaque rupture/thrombosis [7, 8]. Notably, higher levels of Lp(a) increase CVD risk more significantly than would be expected solely from the cholesterol and apoB content contained within it, suggesting a distinct mechanism for Lp(a) in CVD [27, 28]. Potential mechanisms include the carrier role of Lp(a) for oxidized phospholipids, antifibrinolytic effects, and possible pro-platelet effects (Fig. 1) [5, 6, 29–31].

Further laboratory research has identified Lp(a) as a risk factor for thrombosis. Whereas plasminogen activation to plasmin normally results in fibrinolysis of blood clots, Lp(a) does not activate plasminogen due to the inactive protease domain within apo(a) [4, 32]. In vitro and animal studies reveal that apo(a) promotes a pro-thrombotic state by inhibiting fibrinolysis through competitive inhibition of plasminogen conversion [2, 7, 8]. This has been hypothesized to provide a survival benefit by improving wound healing [3, 26]. Some studies in rabbit and human surgical samples suggest that Lp(a) accumulates preferentially at sites of tissue injury, although data on this hypothesis remain sparse [2, 3, 32–34]. The clinical significance of the theoretical antifibrinolytic effect of elevated Lp(a) in humans remains uncertain.

### Observational Studies

Several observational studies have linked Lp(a) to risk for ASCVD (Table 1). The Copenhagen City Heart Study, a large prospective study that assessed the relationship between Lp(a) levels and the risk of MI and ischemic heart disease (IHD), followed 9,330 Danish participants over a 10-year follow-up period and found a stepwise increase in MI and IHD risk with elevated Lp(a) levels. Participants with Lp(a) levels > 120 mg/dL (95th percentile) had an adjusted hazard ratio (HR) for MI of 3.60 (95% CI 1.70–7.70) in women and 3.70 (95% CI 1.70-8.00) in men compared to those with Lp(a) levels < 5 mg/dL (< 22nd percentile) [35]. Additionally, levels > 120 mg/dL were associated with a 3 to 4-fold increase in relative risk (RR) of MI and absolute 10-year risks of 20% and 35% in high-risk women and men, respectively [35].

**Table 1:** Key Observational Studies of Lp(a) and ASCVD Risk

Study	Population at baseline	ASCVD outcome(s)	Adjustments	Key findings	Effect size (HR)
CCHS [26, 35]	9,930 Danish participants without known IHD	MI and IHD	Age HTN DM Other lipids BMI Smoking status Lipid-lowering therapy Menopause, HRT	Extreme Lp(a) levels (> 120 mg/dL or 287 nmol/L) correlated with a three- to four-fold higher risk of MI compared to lower levels of Lp(a) (< 5 mg/dL or 12 nmol/L)	HR 3.60 (95% CI 1.70-7.70) in women HR 3.70 (95% CI 1.70-8.00) in men
ATTICA [36]	1,890 participants without known CVD	Fatal or non-fatal CVD events	Age BMI Smoking status HTN DM Other lipids Statin use CRP LFTs CrCl FH of CVD	Lp(a) levels ≥ 50 mg/dL had increased risk of CVD compared to those with Lp(a) < 50 mg/dL	HR 2.19 (95% CI 1.11-4.28)
UK Biobank [14]	460,506 participants ± baseline ASCVD	Composite of CAD and ischemic stroke	Age Sex Self-reported race	Elevated Lp(a) levels show linear relationship with ASCVD risk per each 50 nmol/L increment Relative to general population, the RR in those with elevated Lp(a) was greater in those without preexisting ASCVD compared to those with preexisting ASCVD	HR of 1.11 (95% CI 1.10-1.12) HR 1.50 (95% CI 1.44-1.56) in those without preexisting ASCVD HR 1.16 (95% CI 1.05-1.27) in those with preexisting ASCVD
MESA [37]	4,565 multi-ethnic participants with well-controlled LDL-C and no ASCVD	Time to composite CHD events	Age Sex Race/ethnicity HTN DM Other lipids BMI Smoking status	Elevated Lp(a) ≥ 50 mg/dL correlated with increased CHD event risk, even with low LDL-C ≤ 100 mg/dL	HR 1.83 (95% CI: 1.02-3.27)
ARIC [16]	13,318 multi-ethnic participants	Incident CVD events	Age Gender Smoking status HTN DM Other lipids	In both Black and White populations, higher Lp(a) levels correlated with increased CVD event risk	HR 1.35 (95% CI 1.06-1.74) in Black population HR 1.27 (95% CI 1.10-1.47) in White population
Mass General Brigham Lp(a) Registry [38]	21,410 participants ± baseline ASCVD	Incident MACE events	Age Sex Self-reported race HTN CKD Other lipids DM and insulin use Smoking status	Elevated Lp(a) > 90^th^ percentile independently increased long-term MACE, regardless of baseline ASCVD	HR 1.21 in those with baseline ASCVD HR 1.93 in those without baseline ASCVD
Women’s Health Study [39]	27,939 healthy women	First MACE event	Age Smoking status DM HTN HRT BMI eGFR	Elevated Lp(a) independently associated with 30-year risk of MACE when adjusted for other covariates	HR 1.33 (95% CI, 1.21-1.47)

*ASCVD *atherosclerotic cardiovascular disease, *BMI *body mass index, *CAD *coronary artery disease, *CHD *coronary heart disease, *CI *confidence interval, *CrCl *creatinine clearance, *CRP *C-reactive protein, *CVD* cardiovascular disease, *DM *diabetes mellitus, *eGFR *estimated glomerular filtration rate, *FH *family history, *HR *hazard ratio, *HTN *hypertension, *IHD *ischemic heart disease, *LFTs *liver function tests, *LDL-C* low-density lipoprotein cholesterol, *Lp(a) *lipoprotein(a), *MACE *major adverse cardiovascular events, *MI *myocardial infarction; RR, relative risk

The ATTICA Cohort Study assessed the 10-year CVD risk in 1,890 healthy individuals from Greece with a combined endpoint of fatal or non-fatal CVD event including acute MI, unstable angina, other identified forms of ischemia, heart failure (HF), chronic arrythmias, or stroke [36]. The study found that those with Lp(a) levels ≥ 50 mg/dL had approximately twice the risk of CVD compared to those with Lp(a) < 50 mg/dL (HR 2.18, 95% CI 1.11–4.28) [<CitationRef CitationID="CR38">38</CitationRef>]. The 10-year CVD event rate was 14% in the group with Lp(a) < 50 mg/dL compared to 24% in the group with Lp(a) ≥ 50 mg/dL. The association remained statistically significant in men (HR 2.00, 95% CI 1.19–2.56) but was not statistically significant in women after adjusting for lipid markers [36].

The UK Biobank prospective study examined the relationship between Lp(a) concentrations and ASCVD risk in a larger cohort of 460,506 middle-aged participants, both with and without a history of ASCVD, over a median follow-up time of 11.2 years. The study highlighted variations in risk across different racial and ethnic groups and emphasized the significance of very high Lp(a) levels (> 150 nmol/L). A linear relationship was found between Lp(a) levels and ASCVD risk with a HR of 1.11 (95% CI 1.10–1.12) for each 50 nmol/L increase in Lp(a) [14]. High Lp(a) concentration (> 150 nmol/L) was more common in individuals with preexisting ASCVD compared to those without (20.3% vs. 12.2%, respectively). However, the RR associated with elevated Lp(a) was more pronounced in those without prior ASCVD (HR 1.50, 95% CI 1.44–1.56) compared to those with prior ASCVD (HR 1.16, 95% CI 1.05–1.27) [14].

The relationship between elevated Lp(a) and the risk of ASCVD in individuals with well-controlled plasma LDL-C levels was analyzed using the Multi-Ethnic Study of Atherosclerosis (MESA) cohort of 4,585 individuals who were all free of ASCVD at recruitment and were not on statin, fibrate, or niacin therapy [37]. The time to coronary heart disease (CHD) events, defined as MI, resuscitated cardiac arrest, and death related to CHD, was recorded over a mean follow up of 13.4 years. Participants were categorized into four groups according to the combination of LDL-C levels (≤ 100 mg/dL vs > 100 mg/dL) and Lp(a) levels (< 50 mg/dL vs ≥ 50 mg/dL). When compared to those with controlled LDL-C and Lp(a) levels, elevated Lp(a) (≥ 50 mg/dL) correlated with significantly increased CHD events, even with optimal LDL-C levels (HR 1.83, 95% CI 1.02–3.27) [37]. However, the study did not show increased risk of CHD in those with elevated LDL-C when Lp(a) was < 50 mg/dL. This study highlights how Lp(a) can contribute to ASCVD risk, even when LDL-C levels are considered optimal.

The Atherosclerosis Risk in Communities (ARIC) study sought to identify risk factors for atherosclerosis and ASCVD in diverse communities. In 3,467 Black individuals and 9,851 White individuals, an increased number of CVD events was seen at 20-year follow-up in those in the highest quintile of Lp(a) levels when compared to the lowest quintile of Lp(a) levels in both the Black population (HR 1.35, 95% CI 1.06–1.74) and White population (HR 1.27, 95% CI 1.10–1.47) [16].

Another study of 16,419 people in Boston, Massachusetts followed over a median time of almost 12 years showed an association between major adverse cardiovascular events (MACE) and Lp(a) levels [38]. In individuals without pre-existing ASCVD, those in the 71-90th and > 90th percentile of Lp(a) levels had a greater than 20% risk of developing MACE (HR 1.21 and 1.26, respectively) when compared to those in the < 50th percentile. In the group with established ASCVD, those in the > 90th percentile of Lp(a) levels had a HR of 1.93 for developing MACE compared to those in the < 50th percentile [38]. This study reiterates that regardless of whether a patient has previously had ASCVD, Lp(a) can play a role in risk for further ASCVD burden. Of course, causality is not clear in observational studies.

Recent evidence from the Women’s Health Study (WHS) strengthens the link between Lp(a) and incident ASCVD. In this large study, the association of high-sensitivity C-reactive protein, LDL-C, and Lp(a) with CVD were studied in 27,939 healthy women over a 30-year period with a composite endpoint of MI, coronary revascularization, stroke, or death from cardiovascular causes [39]. All three biomarkers independently predicted 30-year risk. For Lp(a) specifically, covariate-adjusted HR for the composite endpoint was 1.33 (95% CI 1.21–1.47) [39].

### Mendelian Randomization Studies

Given the strong genetic influence on Lp(a) levels, Mendelian randomization studies have emerged as valuable tools for assessing its causal relationship with ASCVD (Table 2). Large-scale genetic studies support this causal link. In an analysis of 40,486 participants from three Danish cohorts—the Copenhagen City Heart Study, the Copenhagen General Population Study, and the Copenhagen Ischemic Heart Disease Study—genetically determined elevated Lp(a) levels were associated with a 22% increased risk of myocardial infarction (MI) per doubling of Lp(a) levels (HR 1.22, 95% CI 1.09–1.37) [40]. Similarly, a large case-control study involving 6,497 participants (3,145 with coronary artery disease [CAD] and 3,352 controls) from the Precocious Coronary Artery Disease (PROCARDIS) Consortium identified two variants (rs10455872 and rs3798220) in the Lp(a) gene locus (LPA) linked to elevated plasma Lp(a) levels and significantly increased CAD risk (OR 1.70, 95% CI 1.49–1.95; and OR 1.92, 95% CI 1.48–2.49, respectively) [40]. These findings underscore the genetic basis of Lp(a)-mediated cardiovascular risk and provide robust evidence supporting its causal role in ASCVD.

**Table 2:** Genetic and Mendelian Randomization Studies on Lp(a) and ASCVD Risk

Study name	Study design	Population	Genetic variants	ASCVD outcome(s)	Key findings	Effect size (HR/OR)
Kamstrup [35]	Mendelian Randomization	40,486 Danish participants from the CCHS, CGPS, and CIHDS cohorts	Kringle IV type 2 LPA polymorphism	Plasma Lp(a) levels, and MI events	Genetically elevated Lp(a) levels correlated with increased MI risk across all three cohorts	HR 1.22 (95% CI 1.09-1.37)
Clarke [40]	Genetic (Case-Control)	3,145 case participants with CAD and 3,352 control participants from the PROCARDIS cohort	2,100 candidate genes	Plasma Lp(a) levels and risk of CAD	Two LPA gene variants (rs10455872 and rs3798220) correlated with elevated plasma Lp(a) levels The same gene variants correlated with increased CAD risk	OR 1.70 (95% CI 1.49-1.95) OR 1.92 (95% CI 1.48-2.49)
Kyriakou [41]	Genetic (Case-Control)	4,022 cases participants and 4,189 controls from the PROCARDIS cohort	LPA null allele (SNP rs41272114)	Plasma Lp(a) levels and risk of CAD	LPA null allele correlated with decreased Lp(a) levels and decreased CAD risk	OR 0.79 (95% CI 0.66-0.97)
Lim [42]	Mendelian Randomization	3,000 Finnish and 3,000 non-Finnish European participants	3,000 whole-exome sequences	Plasma Lp(a) levels and risk of CVD	Five spliced variants of the LPA locus that correlated with lowered Lp(a) levels conferred cardiovascular protection	OR 0.84 (95% CI 0.80-0.88)
Burgess (EPIC-CVD Consortium) [43]	Mendelian Randomization	48,333 participants of European descent from five studies	Multiple LPA gene variants	Presence of CHD	Every 10 mg/dL decrease in Lp(a) concentration correlated with a 5.8% CHD risk reduction	OR 0.94 (95% CI 0.93-0.95)

*CAD *coronary artery disease, *CCHS *Copenhagen City Heart Study, *CGPS *Copenhagen General Population Study, *CHD *coronary heart disease, *CI *confidence interval, *CIHDS *Copenhagen Ischemic Heart Disease Study, *CVD *cardiovascular disease, *EPIC *European Prospective Investigation into Cancer and Nutrition, *HR *hazard ratio, *Lp(a)* lipoprotein(a), *LPA *lipoprotein(a) gene locus, *MI *myocardial infarction, *OR *odds ratio, *PROCARDIS* Precocious Coronary Artery Disease Consortium, *SNP* single-nucleotide polymorphism

Lower Lp(a) levels have also been shown to have a protective effect. A Mendelian randomization study of a large, mostly Finnish population found that five splice variants at the LPA locus leading to lower Lp(a) concentrations were associated with a lower risk of CVD (OR 0.84, 95% CI 0.80–0.88) [42]. Analysis of the PROCARDIS Consortium also showed that a null allele at the LPA locus (found in about 3% of the population) was associated with a lower risk of CAD (OR 0.79, 95% CI 0.66–0.97) when compared to non-carriers [41]. Most data on CVD risk come from individuals with high Lp(a) levels so this inverse finding points to the causal effect of Lp(a) as a risk factor for CVD and provides hope that Lp(a)-lowering therapies may reduce that risk.

A Mendelian randomization analysis of the European Prospective Investigation into Cancer and Nutrition (EPIC)-CVD Consortium of 62,240 patients with CHD and 127,299 controls redemonstrated the relationship between absolute change in genetically predicted Lp(a) concentration and CHD risk [43]. Every 10 mg/dL decrease in Lp(a) concentration was associated with a 5.8% lower CHD risk (OR 0.94, 95% CI 0.93–0.95) [43]. This reduction in risk was less than the equivalent change in LDL-C, suggesting a greater magnitude of reduction would be necessary for Lp(a)-lowering therapies to be effective [43].

### Meta-Analyses

Several meta-analyses have highlighted associations between Lp(a) concentrations and ASCVD risk, reinforcing the findings of individual studies (Table 3). In 2009, the Emerging Risk Factors Collaboration examined the relationship between Lp(a) levels and the risk of developing CHD or ischemic stroke among primary prevention populations [44]. This analysis pooled data from 126,634 participants without a history of CHD or stroke across 36 prospective cohorts. It confirmed Lp(a) as an independent, albeit modest, risk factor for incident nonfatal MI and death from CHD, with an adjusted risk ratio of 1.13 (95% CI 1.09–1.18) [44]. A higher risk was observed in participants with Lp(a) levels in the highest quartile [44]. The study also found a trend linking increased Lp(a) levels and cardiovascular events in patients who had elevated non–high-density lipoprotein cholesterol (non-HDL-C) levels [44]. While most participants were White, the study included various ethnic/racial groups, and no significant differences in risk estimates were observed across these groups.

**Table 3:** Meta-Analyses on Lp(a) and ASCVD Outcomes

Meta-analysis	Sample size	Population	ASCVD outcome(s)	Key findings	Effect size (HR/OR/RR)
Emerging Risk Factors Collaboration [44]	36 studies totaling 126,634 participants	Participants with baseline Lp(a) measurements	CVD, CHD, MI, or stroke events	Lp(a) was an independent risk factor for incident nonfatal MI and death from CHD	HR 1.13 (95% CI 1.09-1.18)
Erqou [45]	40 studies totaling 11,396 participants and 46,938 controls	Participants with recorded Lp(a) isoforms	CHD (defined by MI, angina, coronary stenosis, or revascularization) or ischemic stroke events	Smaller apo(a) isoforms were associated with a two-fold increase in the risk of CHD or ischemic stroke events	RR 2.08 (95% CI 1.67-2.58) for CHD events RR 2.14 (95% CI 1.85-2.97) for ischemic stroke events
O’Donoghue [46]	11 studies, including the PEACE, CARE, and PROVE IT-TIMI 22 trials, totaling 18,978 participants	Participants with either stable CAD or after ACS	MACE events defined by the composite of CVD-related death, MI, or stroke	In secondary prevention population, the highest quintile of Lp(a) levels correlated with increased risk of MACE When LDL-C was elevated, higher Lp(a) levels were significantly associated with MACE	OR 1.40 (95% CI 1.15-1.71) OR 1.46 (95% CI 1.23-1.73)
Willeit [47]	7 randomized, placebo-controlled trials of statin therapy totaling 29,069 participants	Participants on statin therapy with baseline and repeat Lp(a) measurements	CV events, defined as fatal or non-fatal CHD, stroke, or revascularization procedures	Lp(a) levels had a linear relationship with CV risk, even when LDL-C is controlled on statin therapy	HR 0.94 (0.81-1.10) for Lp(a) of 15-30 mg/dL HR 1.06 (0.94-1.21) for Lp(a) of 30-50 mg/dL HR 1.43 (1.15-1.76) for Lp(a) of 50 mg/dL or higher
Bhatia [48]	6 randomized, placebo-controlled trials of statin therapy from the Lipoprotein(a) Studies Collaboration totaling 27,658 participants	Participants with baseline Lp(a) and LDL-C measurements	Fatal or nonfatal CHD, fatal or nonfatal stroke, or any coronary or carotid revascularization	Among statin-treated participants, Lp(a) levels > 50 mg/dL correlated with increased risk across all quartiles of achieved LDL-C level and absolute change in LDL-C level, even in the lowest LDL-C quartile The greatest risk was observed when both Lp(a) level > 50 mg/dL and LDL-C level in the highest quartile	HR 1.38 (95% CI, 1.06-1.79) HR 1.90 (95% CI, 1.46-2.48)
Wong [49]	5 prospective studies comprising a multi-ethnic cohort totaling 27,756 people	Participants with baseline Lp(a) measurements without previous ASCVD	Nonfatal and fatal MI and stroke, revascularization, and CHD death	Lp(a) levels above the 50^th^ percentile correlated with incremental increased in risk in an independent manner.	HR 1.06 (95% CI: 0.99-1.14) for Lp(a) in the 50-75^th^ percentiles HR1.18 (95% CI: 1.09-1.28 for Lp(a) in the 75-90^th^ percentiles HR 1.46 (95% CI: 1.33-1.59) for Lp(a) in the ≥ 90^th^ percentile

*ACS *acute coronary syndrome, *ASCVD* atherosclerotic cardiovascular disease, *CAD* coronary artery disease, *CHD* coronary heart disease, *CI* confidence interval, *CV* cardiovascular, *CVD* cardiovascular disease, *HR* hazard ratio, *LDL-C* low density lipoprotein cholesterol, *Lp(a)* lipoprotein(a), *MACE* major adverse cardiovascular events, *MI* myocardial infarction, *OR* odds ratio, *RR* relative risk

Given the unclear clinical implications of the relationship between Lp(a) concentration and risk of ASCVD, one study sought to further clarify the impact of Lp(a) subtypes, specifically focusing on the size of apo(a) isoforms [45]. They hypothesized that smaller isoforms represent a stronger risk factor which is based on the theory that smaller isoforms are more pathogenic due to increased binding of oxidized phospholipids, higher tendency to accumulate in blood vessel walls secondary to enhanced lysine-binding and fibrin interactions, and stronger thrombogenic effect from greater inhibition of plasmin activity [50–53]. They analyzed 40 studies published between 1970 and 2009 that reported on the relationship between apo(a) isoforms and the risk of CVD and ischemic stroke, encompassing over 58,000 participants. Their analysis revealed that smaller apo(a) isoforms were associated with a two-fold increase in the risk of CHD (RR 2.08, 95% CI 1.67-2.58) and ischemic stroke (RR 2.14, 95% CI 1.85-2.97) [45]. This analysis did not determine whether isoform size was an independent risk factor, separate from Lp(a) concentration and other cardiovascular risk factors.

A 2014 paper analyzed Lp(a) levels as a prognostic biomarker for secondary prevention in individuals with CAD [46]. Plasma levels were measured in 6,708 participants with CAD from three cohorts (PEACE, CARE, and PROVE IT–TIMI 22 trials), and the data were then combined with eight previously published studies, totaling 18,978 participants. Within the three cohorts, Lp(a) levels were not associated with MACE when modeled as a continuous variable (OR 1.03 per SD, 95% CI 0.96–1.11) or by quintile (Q5:Q1 OR 1.05, 95% CI 0.83–1.34) [46]. However, when the data were combined with the other eight studies, Lp(a) levels in the highest quintile correlated with an increased risk of MACE (OR 1.40, 95% CI 1.15–1.71), although there was notable heterogeneity between studies [46]. Furthermore, a significant association was found between Lp(a) levels and MACE in those with LDL-C levels ≥ 130 mg/dl (OR 1.46, 95% CI 1.23–1.73), while no significant association was found in those with LDL-C levels < 130 mg/dl (OR 1.20, 95% CI 0.90–1.60) [46]. This meta-analysis underscored the relationship between Lp(a) levels and ASCVD in a secondary prevention population and the potential additive interplay between LDL-C and Lp(a).

Several other meta-analyses have highlighted the relationship between LDL-C and Lp(a) levels in relation to ASCVD risk. A 2018 meta-analysis of seven randomized, placebo-controlled statin trials, involving 29,069 participants, indicated a linear relationship between Lp(a) levels and cardiovascular risk, even among patients on statin treatment [47]. This meta-analysis supports the notion of Lp(a) as an independent risk factor, consistent with other recent studies showing similar results [36, 47]. Further insights were provided by a 2024 participant-level meta-analysis of six placebo-controlled statin trials, involving 27,658 participants, which emphasized the independent and additive relationship between LDL-C and Lp(a) levels [48]. In statin-treated participants, an Lp(a) level > 50 mg/dL was associated with an increased risk of ASCVD across all quartiles of LDL-C levels, including those in the lowest quartile of achieved LDL-C levels (< 77 mg/dL) (HR 1.38, 95% CI 1.06–1.79) [48]. The greatest risk was observed when both Lp(a) and LDL-C levels were in the highest quartile (HR 1.90, 95% CI 1.46–2.48), indicating an additive effect [48].

A 2024 analysis that pooled several large cohorts, including MESA, ARIC, Coronary Artery Risk Development in Young Adults (CARDIA), Jackson Heart Study (JHS), and Framingham Heart Study-Offspring (FHS-OS), encompassing 27,756 people, showed that higher Lp(a) levels are associated with an increased risk of ASCVD. Risk increased in a dose-dependent fashion with increasing risk at serum levels in the 50-75th percentile (HR 1.06, 95% CI 0.99–1.14), 75-90th percentile (HR 1.18, 95% CI 1.09–1.28) and > 90th percentile (HR 1.46, 95% CI 1.33–1.59) [49].

## Lp(a) Risk Factor for Aortic Stenosis

Multiple studies have demonstrated a connection between Lp(a) levels and the development of CAVS. A genome-wide study of the Cohorts for Heart and Aging Research in Genomic Epidemiology (discovery population) along with an independent cohort of patients with CAVS identified a specific single-nucleotide polymorphism (SNP) at the Lp(a) gene locus that predicted Lp(a) levels and was associated with CAVS and the need for aortic valve replacement (AVR) [54]. These findings were replicated across multiple ethnic groups. Additionally, an analysis of the Copenhagen City Heart Study showed that Lp(a) levels were associated with increasing hazard ratios for CAVS, with levels > 90 mg/dL carrying nearly a threefold increased risk (HR 2.90, 95% CI 1.80–4.90) compared to those with Lp(a) levels < 5 mg/dL [55].

In the EPIC-Norfolk study, a particular genetic variant linked to high Lp(a) levels demonstrated an increased risk for CAVS with a HR of 1.78 (95% CI 1.11–2.87) for heterozygous carriers and a HR of 4.83 (95% CI 1.77–13.20) for homozygotes [56]. Participants in the highest tertile of measured Lp(a) levels were found to be at higher risk for developing CAVS when compared to those in the lowest tertile (HR 1.57, 95% CI 1.02–2.42) [56]. Even among asymptomatic patients with familial hypercholesterolemia (FH), elevated Lp(a) levels were independently associated with CAVS, with each 10 mg/dL increase in Lp(a) associated with an 11% higher risk of aortic valve calcification (OR 1.11, 95% CI 1.01–1.20) [57]. A large electronic health record case-control study of 44,703 patients in the Genetic Epidemiology Research on Aging cohort in the Kaiser Permanente system found that genetic variants in the Lp(a) gene were linked to an increased likelihood of severe CAVS and the need for AVR [58]. The highest-risk alleles were associated with more than a two-fold increased risk of developing CAVS, with odds ratios of 2.05 (95% CI 1.37–3.07) for rs10455872 homozygotes, 3.74 (95% CI 1.03–13.62) for rs3798220 homozygotes, and 2.00 (95% CI 1.17–3.44) for compound heterozygotes [58].

Furthermore, a prospective cohort study of patients with mild-to-moderate CAVS revealed that those in the highest tertile of Lp(a) levels experienced a faster rate of CAVS progression [59]. The study also suggested a potential synergistic effect between oxidized phospholipid (OxPL) levels and Lp(a) in the progression of disease [59]. A recent meta-analysis showed OxPL-carrying lipoproteins (OxPL-apoB), including Lp(a), were associated with faster progression of CAVS when measured by peak aortic jet velocity [60]. The same analysis also showed that both OxPL-apoB and Lp(a) levels were associated with the development of CAVS at 9.5-year follow-up with odds ratios of 1.19 (95% CI 1.07–1.32) and 1.13 (95% CI 1.01–1.27), respectively [60].

## Lp(a) as a Risk Factor for Stroke, Peripheral Arterial Disease, and Heart Failure

Lp(a) has also been associated with other cardiovascular disorders including ischemic stroke, peripheral arterial disease (PAD), and HF. Several studies have shown a link between Lp(a) and ischemic stroke [43, 60–62]. However, much of the significant evidence shows a weak association, and overall, findings are mixed [43, 60]. An early prospective cohort study showed that elevated Lp(a) levels were associated with a risk ratio of 1.10 (95% CI 1.02–1.18) for developing ischemic stroke after adjusting for other risk factors [44]. An analysis of the Copenhagen City Heart Study found that participants with Lp(a) levels > 50 mg/dl had a multivariable-adjusted HR of 1.20 (95% CI 1.13–1.28) for ischemic stroke. An analysis using SPARCL data of 2,814 stroke survivors showed no association between Lp(a) levels and recurrent strokes [63]. Interestingly, data in children have shown a more consistent association between Lp(a) and ischemic stroke [64]. More data are needed, particularly on whether emerging Lp(a) targeting therapies influence the incidence of primary or secondary ischemic stroke.

Large-scale genetic studies have further linked Lp(a) to both PAD and HF. One Mendelian randomization study of multiple cohorts found an association between LPA genetic variants and PAD, as measured by ankle-brachial indices (ABI) (OR 1.65) [65]. Another study showed a 16% increased risk of femoral atherosclerotic disease, as measured by ABI, in individuals with similar genetic variants [66]. Also, a systematic review (8 studies including 73,410 patients) concluded a positive relationship between Lp(a) and HF was likely, but that more research was needed to further elucidate the relationship [67].

## Clinical Implications of Lp(a) Reduction

Given the wealth of evidence linking elevated Lp(a) levels to CVD, interventions to reduce Lp(a) levels could potentially offer significant risk reduction. One study using data from the UK Biobank found that among participants with Lp(a) levels above 175 nmol/L, an 80% reduction in Lp(a) was estimated to reduce the risk of composite fatal and non-fatal CVD by 23.1% in the general population and by 20.0% in those with established CVD [68]. CVD was defined in the study as a composite of fatal and non-fatal cardiovascular events, which included CHD, peripheral vascular disease, and CAVS. Several Mendelian randomization studies have estimated the level of Lp(a) reduction necessary to reduce the risk of CVD by an equivalent amount as a 38.67 mg/dL reduction of LDL-C [41, 47, 67]. These estimates vary from 50 mg/dL to 101.5 mg/dL, so in order to reduce CVD risk associated with high circulating Lp(a) levels, a large treatment effect would be necessary [41, 47, 67].

Existing lipid-lowering therapies have only modest effects on Lp(a) and are often confounded by the concurrent reduction of LDL-C. As discussed above, the risks associated with LDL-C and Lp(a) appear additive and independent of each other, with Lp(a)-mediated risk persisting even in patients undergoing LDL-C-lowering therapies [48]. There have been a few clinical trials that lowered Lp(a) using existing therapies. Data from the Heart Protection Study 2-Treatment of HDL to Reduce the Incidence of Vascular Events (HPS2-THRIVE) Study comparing niacin-laropiprant and simvastatin to placebo and simvastatin showed that niacin-laropiprant reduced Lp(a) levels by about 30%, but also that an approximately 40% reduction would be necessary to see clinically significant benefits [69]. The ODYSSEY OUTCOMES trial, which compared alirocumab to placebo, was able to show that Lp(a) reduction independently contributed to a reduced incidence of MACE; each 5 mg/dL reduction of Lp(a) correlated with a 2.5% reduction in MACE [70]. These data only support a potential benefit of lowering Lp(a) and further results on CVD risk from ongoing studies using more focused Lp(a)-lowering therapies are awaited.

Novel therapies targeting Lp(a) are still undergoing clinical trials, but early data are promising. There are already robust data on how significant reductions in Lp(a) could improve long-term cardiovascular outcomes. Ongoing research will help clarify these effects.

## Algorithm for Lp(a) Screening and Management

The 2018 American Heart Association (AHA)/American College of Cardiology (ACC) Blood Cholesterol guideline identifies Lp(a) as a risk enhancer that can be used to refine risk in borderline and intermediate ASCVD risk individuals [71]. The 2024 NLA guidelines, 2022 European Atherosclerosis Society and 2021 Canadian Cardiology Society guidelines, recommend Lp(a) be tested at least once in all adults for risk stratification purposes [9, 70, 71]. Individuals with Lp(a) levels greater than 125 nmol/L or 50 mg/dL are considered high risk, while those with levels between 75–125 nmol/L or 30–50 mg/dL are categorized as intermediate risk. Since Lp(a) levels are stable over time and no currently approved treatments meaningfully lower Lp(a) levels, recommendations are for a one-time measurement [72].

Due to its genetic basis, screening is strongly encouraged for first-degree relatives of individuals with elevated Lp(a) levels, as well as those with a family history of premature CVD or FH [9, 71]. Cascade testing is recommended even for pediatric patients with first-degree relatives who have high-risk Lp(a) levels or FH [70, 73]. There is some evidence suggesting that elevated Lp(a) levels in childhood may predict early ASCVD, which could justify future screening in pediatric populations [74]. Although no effective treatments are currently approved, identifying individuals with elevated Lp(a) through screening can identify patients in which to prioritize aggressive risk factor management due to their higher baseline risk [10]. Additionally, this screening can identify potential candidates for emerging treatments once they become available.

## Conclusion

The atherogenic properties of Lp(a) are thought to significantly contribute to the initiation and progression of CVD. Numerous studies have demonstrated a strong association between elevated Lp(a) levels and an increased risk of ASCVD and CAVS, with weaker but notable associations observed with heart failure and ischemic stroke. Despite the well-established risk posed by elevated Lp(a), no currently approved and available therapies specifically target and significantly reduce Lp(a) levels. Existing guidelines advocate for one-time Lp(a) screening in adults and recommend managing elevated levels through aggressive control of standard modifiable cardiovascular risk factors. However, several emerging pharmacological therapies designed to lower Lp(a) are currently under investigation and hold promise for reducing cardiovascular risk. Ongoing cardiovascular outcome trials will be essential in further solidifying the causal role of Lp(a) in CVD and guiding future therapeutic strategies.

## Key References

- Ridker PM, Moorthy MV, Cook NR, Rifai N, Lee IM, Buring JE. Inflammation, Cholesterol, Lipoprotein(a), and 30-Year Cardiovascular Outcomes in Women. *N Engl J Med*. Published online August 31, 2024. 10.1056/NEJMoa2405182.
  - This study highlights the long-term, longitudinal association of Lp(a) and cardiovascular disease in primary prevention.
- Burgess S, Ference BA, Staley JR, et al. Association of LPA Variants With Risk of Coronary Disease and the Implications for Lipoprotein(a)-Lowering Therapies: A Mendelian Randomization Analysis. *JAMA Cardiol*. 2018;3(7):619-627. 10.1001/jamacardio.2018.1470.
  - In this mendelian randomization analysis, CHD risk determined by genetically predicted Lp(a) was proportional to changes in plasma Lp(a) concentration.
- Bhatia HS, Dweck MR, Craig N, et al. Oxidized Phospholipids and Calcific Aortic Valvular Disease. *J Am Coll Cardiol*. 2024;84(25):2430-2441. 10.1016/j.jacc.2024.08.070.
  - In this analysis, Lp(a) and oxidized phospholipids were associated with aortic valve calcium and aortic valve max velocity progression.
- Koschinsky ML, Bajaj A, Boffa MB, et al. A focused update to the 2019 NLA scientific statement on use of lipoprotein(a) in clinical practice. *J Clin Lipidol*. 2024;18(3):e308-e319. 10.1016/j.jacl.2024.03.001.
  - National Lipid Association statement highlighting the need for at least once Lp(a) measurement in all adults.

## Data Availability

No datasets were generated or analysed during the current study.
